# Multimorbidity and catastrophic health expenditure: Evidence from the China Health and Retirement Longitudinal Study

**DOI:** 10.3389/fpubh.2022.1043189

**Published:** 2022-10-26

**Authors:** Haofei Li, Enxue Chang, Wanji Zheng, Bo Liu, Juan Xu, Wen Gu, Lan Zhou, Jinmei Li, Chaojie Liu, Hongjuan Yu, Weidong Huang

**Affiliations:** ^1^Department of Health Economics, School of Health Management, Harbin Medical University, Harbin, China; ^2^Heilongjiang Medical Service Management Evaluation Center, Harbin, China; ^3^School of Psychology and Public Health, La Trobe University, Melbourne, VIC, Australia; ^4^Department of Hematology, The First Affiliated Hospital, Harbin Medical University, Harbin, China

**Keywords:** multimorbidity, catastrophic health expenditure, elderly, China, economic burden

## Abstract

**Background:**

Population aging accompanied by multimorbidity imposes a great burden on households and the healthcare system. This study aimed to determine the incidence and determinants of catastrophic health expenditure (CHE) in the households of old people with multimorbidity in China.

**Methods:**

Data were obtained from the China Health and Retirement Longitudinal Study (CHARLS) conducted in 2018, with 3,511 old people (≥60 years) with multimorbidity responding to the survey on behalf of their households. CHE was identified using two thresholds: ≥10% of out-of-pocket (OOP) health spending in total household expenditure (THE) and ≥40% of OOP health spending in household capacity to pay (CTP) measured by non-food household expenditure. Logistic regression models were established to identify the individual and household characteristics associated with CHE incidence.

**Results:**

The median values of THE, OOP health spending, and CTP reached 19,900, 1,500, and 10,520 Yuan, respectively. The CHE incidence reached 31.5% using the ≥40% CTP threshold and 45.6% using the ≥10% THE threshold. It increased by the number of chronic conditions reported by the respondents (aOR = 1.293–1.855, *p* < 0.05) and decreased with increasing household economic status (aOR = 1.622–4.595 relative the highest quartile, *p* < 0.001). Hospital admissions over the past year (aOR = 6.707, 95% CI: 5.186 to 8.674) and outpatient visits over the past month (aOR = 4.891, 95% CI: 3.822 to 6.259) of the respondents were the strongest predictors of CHE incidence. The respondents who were male (aOR = 1.266, 95% CI: 1.054 to 1.521), married (OR = 1.502, 95% CI: 1.211 to 1.862), older than 70 years (aOR = 1.288–1.458 relative to 60–69 years, *p* < 0.05), completed primary (aOR = 1.328 relative to illiterate, 95% CI: 1.079 to 1.635) or secondary school education (aOR = 1.305 relative to illiterate, 95% CI: 1.002 to 1.701), lived in a small (≤2 members) household (aOR = 2.207, 95% CI: 1.825 to 2.669), and resided in the northeast region (aOR = 1.935 relative to eastern, 95% CI: 1.396 to 2.682) were more likely to incur CHE.

**Conclusion:**

Multimorbidity is a significant risk of CHE. Household CHE incidence increases with the number of reported chronic conditions. Socioeconomic and regional disparities in CHE incidence persist in China.

## Introduction

The World Health Organization (WHO) defined the presence of two or more chronic conditions in the same patient as multimorbidity (1). The prevalence of multimorbidity increases by age, which is deemed as a great challenge for an aging society (2, 3). China has the largest population of old people in the world (4). By the end of 2021, the population aged 60 years and above had accounted for 18.9% (14.2% for those aged 65 years and above) of the total population in China (5). China entered into an aged society at an unprecedented speed (4). This is accompanied by rapid increase in the prevalence of chronic conditions (6). Coexistence of multiple chronic conditions is common in old populations (7). The estimated prevalence of multimorbidity in people over 60 years ranges from 6.4 to 76.5% (2.5–54.9% with three or more chronic conditions) in China due to heterogeneity of study methods (8). A study found that more than 60% of urban elderly over 70 years lived with multimorbidity, higher than that in some other countries (9).

Multimorbidity is an important cause of limitations in activities of daily life and declined quality of life in old people (2, 10). It is also associated with increased risk of death and economic loss (11, 12). The increased prevalence of age-related diseases (6), in particular multimorbidity, has a profound impact on the financial burden of the households and the society at large (12–15). Multimorbidity increases use of healthcare services at both outpatient and inpatient settings (9, 16). A study in Brazil found that patients with multimorbidity use medical services twice as much as those without multimorbidity (17). There exists a positive correlation between the number of chronic conditions and out-of-pocket (OOP) health spending (9, 16, 18). It was estimated that multimorbidity increases OOP health spending by 13% in Mexico (19). An Australian study reported that each additional chronic condition can increase the likelihood of severe financial burden due to healthcare by 46% (18).

Health expenditure is considered catastrophic once it jeopardizes the ability of a household to maintain usual standards of living (20). Catastrophic health expenditure (CHE) threatens the ability of the household to purchase other goods and services, potentially driving the household into poverty (21, 22). Previous studies have shown that even in the wealthy households with health insurance coverage, multimorbidity is significantly associated with the occurrence of CHE (12, 23). According to the WHO, global health spending is rising rapidly, and this trend is particularly profound in the low- and middle-income countries (LMICs) (24). The high-income countries have experienced an average annual growth of health expenditure of 4%, compared with 6% in the LMICs (24). Most LMICs still rely heavily on OOP payments for healthcare, despite certain progress in health insurance development (24, 25). China is one of the examples. Although China has achieved almost universal coverage of social health insurance, it provides limited financial protection for enrollees due to limited funding pools (13, 26). The OOP health spending as a proportion of total health expenditure in China has remained higher than the reasonable level defined by the WHO (27).

There is a paucity in the literature documenting the association between multimorbidity and CHE in China, despite extensive studies into the use and burden of healthcare services for individuals living with multimorbidity (9, 12, 28). Zhou et al. estimated that the incidence of CHE in the single elderly with multimorbidity in rural Shandong can be as high as 64.2% (29). Fu et al. identified a CHE incidence of 56.6% in the households with diabetic patients with multimorbidity in China (23). This study aimed to address the gap in the literature by determining the CHE incidence and its determinants in the households of old people in China with multimorbidity using a national representative dataset.

## Methods

### Data source

Data were extracted from the China Health and Retirement Longitudinal Study (CHARLS), a nationally representative longitudinal survey of Chinese people over the age of 45 years. The CHARLS started in 2011, collecting information regarding the sociodemographic characteristics of respondents and their health-related behaviors, health status, health insurance, and health services utilization (30). Study participants were recruited using a probability-proportional-to-size (PPS) sampling strategy across 28 provinces/regions in China. Data were collected through face-to-face computer-assisted personal interviews (30). The CHARLS obtained ethical approval from the Biomedical Ethics Review Committee of Peking University (IRB0000105IRB00001052–11015).

In this study, we used the data from the fourth wave of CHARLS conducted in 2018–2019. Eligible study participants included those who were 60 years or older, reported two or more chronic non-communicable diseases, and had a complete record (no missing values). This resulted in a final sample of 3,511 households, with one respondent for each household, for data analyses.

### Measurements

#### Multimorbidity

Multimorbidity was defined as coexistence of two or more chronic non-communicable diseases (1). In the CHARLS, respondents were asked to report 14 chronic conditions diagnosed by a medical doctor, including hypertension, dyslipidemia, diabetes, cancer or malignant tumor, chronic lung diseases, liver diseases, heart attack, stroke, kidney diseases, stomach and other digestive diseases, emotional, nervous and psychiatric problems, memory-related diseases, arthritis and rheumatism, and asthma. We counted the number of chronic conditions reported by each respondent.

#### Catastrophic health expenditure

There is currently no uniform threshold for defining catastrophic health expenditures (CHE), and there are two thresholds most commonly used in academia: the percentage (≥ 10%) of health expenditure paid out of pocket (OOP) in total household expenditure (THE) (21, 31, 32) and OOP health spending as a percentage (≥ 40%) of household capacity to pay (CTP) measured by non-food expenditure (14, 33–35). We set CHE as a binary variable:


$$CHE=\{\begin{array}{c} 0\;i\text{f}<\text{ threshold}\\ \text{1 }i\text{f}\geq \text{threshold} \end{array}$$

OOP counted all out-of-pocket health spending of the respondent and his/her spouse (12, 13) including those for outpatient visits, hospital admissions, and medications over the past year.

CTP was calculated as household expenditure over the past year excluding the subsistence need for foods (12, 13), which covered communication (post, internet, telephone and cell phone), utility (water, electricity), fuel (gas, petrol, coal), local transportation (including parking), domestic help (babysitting, housekeeping, personal care), entertainment (printing materials, audios and videos, cinema, bars, travels), daily necessities (toiletries, kitchen supplies, clothing and bedding), restaurants and banquets, education and training, OOP health expenses (including direct and indirect medical expenses and no including portion paid by Medicare), fitness and beauty (make-up products, beauty salons, gym, massage), rents, maintenance and repairment (house, vehicle, appliance, communication products), governmental tax and fee, donations, and household items (furniture, decorative items, durable electronics, automobiles).

#### Household and respondent characteristics

The variables measuring household characteristics included geographic location (eastern, central, western, and northeastern), residency (urban, rural), household size (number of cohabited family members), and economic status. Overall, the eastern region is most developed while the western is least developed in mainland China (36). There is also a significant urban-rural disparity in socioeconomic development (37). Household economic status was measured using per capita non-health expenditure, which was subsequently divided into quartiles.

The respondent characteristics were measured by sex (male, female), age (years), marital status (married, others), educational attainment (illiterate, primary school, secondary school, university), number of chronic conditions (2, 3, 4, ≥5), hospital admissions over the past year (yes, no), outpatient visits over the past month (yes, no), and coverage (yes, no) of different types of health insurance. There were three major types of voluntary social health insurance programs: basic medical insurance for urban employees, basic medical insurance for urban residents, and new rural cooperative medical scheme. In some municipalities, a unified basic medical insurance program was established for both urban and rural residents.

### Statistical analysis

Household and respondent characteristics were described using frequency distributions. Both mean and median (interquartile) values for household expenditure, OOP health spending, and CTP were presented.

We further explored the determinants of CHE with OOP not < 40% of CTP as the criterion of catastrophic health expenditure. Because this threshold removes the rigid impact of household food expenditures, it can better avoid deviations in the measurement of catastrophic health expenditures for low-income households (14). The incidence of CHE was calculated and compared among the respondents with different individual and household characteristics through Chi-square tests. Multivariate logistic regression models were established to determine the predictors of CHE, as indicated by the adjusted odds ratio (aOR) with 95% confidence interval. The discriminatory validity and calibration ability of the logistic regression models were tested using the area under the ROC curve (AUC > 0.75) and the Hosmer-Lemeshow Goodness-of-Fit (between the predicted value and the actual observed value) test (*p* > 0.05), respectively (38).

All statistical analyses were performed using SPSS 26.0 (IBM Corporation). A two-side *p*-value of < 0.05 was considered statistically significant.

## Results

### Characteristics of study participants

The respondents had an average age of 69.37 (SD = 7.16) years: 58.4% were younger than 70 years. Just over half (53.0%) were male and 28.40% were illiterate. Most respondents were married (75.0%) at the time of the survey, were enrolled in the new rural cooperative medical scheme (63.8%), and reported more than two chronic conditions (64.8%). About one in eleven (11.3%) were admitted to a hospital over the past year and 11.4% visited outpatient clinics over the past month.

The vast majority of the households of the respondents were located in rural areas (77.2%) and had no more than two household members (69.1%). More than one third (34.8%) were sampled from the least developed western region (Table 1).

**Table 1:** Sociodemographic characteristics of study participants with multimorbidity (*n* = 3511).

**Characteristics**	***n***	**%**
Sex		
Male	1862	53.0
Female	1649	47.0
Age (Years)		
60–69	2051	58.4
70–79	1088	31.0
≥80	372	10.6
Marital status		
Married	2635	75.0
Else	876	25.0
Education		
Illiterate	997	28.4
Primary school	1590	45.3
Secondary school	872	24.8
University	52	1.5
Number of chronic diseases		
2	1236	35.2
3	903	25.7
4	611	17.4
≥5	761	21.7
Hospital admissions over the past year		
Yes	395	11.3
No	3116	88.7
Outpatient visits over the past month		
Yes	399	11.4
No	3112	88.6
Health insurance		
Basic medical insurance for urban employees	502	14.3
Basic medical insurance urban and rural residents	425	12.1
New rural cooperative medical scheme	2241	63.8
Basic medical insurance for urban residents	157	4.5
Others	82	2.3
None	104	3.0
Residency		
Rural	2712	77.2
Urban	799	22.8
Household size		
≤2	2426	69.1
>2	1085	30.9
Geographic location		
Eastern	983	28.0
Central	1034	29.5
Western	1223	34.8
Northeastern	271	7.7

### Catastrophic health expenditure

The household expenditure data were positively skewed. The median values of THE, OOP health spending, and CTP reached 19,900, 1,500, and 10,520 Yuan, respectively. The incidence of CHE was 45.6% using the threshold of ≥10% THE, compared with 31.5% using the threshold of ≥40% CTP. It increased with the number of chronic conditions and decreased with rising household economic status. Although the households with higher economic status (measured by per capita non-health expenditure) spent more on medical care, their incidence of CHE were lower. Similarly, urban households had lower incidence of CHE, despite spending more on medical care (Table 2).

**Table 2:** Household burden of out–of–pocket (OOP) payment for medical expenditure and catastrophic health expenditure (CHE) of study participants (*n* = 3511).

**Indicator**	**Number of chronic conditions reported by respondents**				**Household economic status (per capita non-health** **expenditure)**				**Residency**		**Total**
	**2**	**3**	**4**	**≥5**	**Q1 (Lowest)**	**Q2**	**Q3**	**Q4 (Highest)**	**Rural**	**Urban**	
**OOP medical expenditure**											
Mean	4352	5427	7199	11495	5173	6635	6539	8344	5950	9121	6672
Median (P25, P75)	720 (0, 3240)	1440 (120, 4800)	1800 (288, 6000)	3600 (930, 9640)	1200 (24, 4800)	1560 (240, 5400)	1440 (96, 5520)	2040 (36, 6550)	1440 (125, 4800)	1782 (0, 7000)	1500 (120, 5400)
**Capacity to pay**											
Mean	20634	21711	22719	24500	7861	13113	19180	48343	19142	32191	22112
Median (P25, P75)	9350 (4356, 18790)	10620 (5060, 22540)	10920 (4860, 21900)	12140 (5602, 26518)	4370 (2196, 7900)	8560 (4868, 14320)	12200 (7048, 21885)	28060 (13571, 49148)	8739 (4300, 18305)	18000 (9720, 33840)	10520 (4900, 22028)
**Household total expenditure**											
Mean	31526	32927	33121	36092	9515	19399	30810	72973	28011	50610	33154
Median (P25, P75)	18018 (8863, 34465)	20100 (9600, 38520)	20283 (10040, 37050)	22672 (10636, 41531)	5976 (3256, 10500)	14100 (9970, 22400)	23124 (17370, 34415)	50820 (34330, 80260)	16376 (8080, 30605)	34560 (21070, 56500)	19900 (9672, 37520)
**CHE Incidence (%)**											
10% household expenditure	35.4	43.5	50.2	61.1	60.8	51.4	40.4	29.9	47.6	38.9	45.6
40% capacity to pay	23.6	29.5	35.4	43.8	44.5	35.5	27.1	19.0	33.1	26.2	31.5

**Figure 1:**
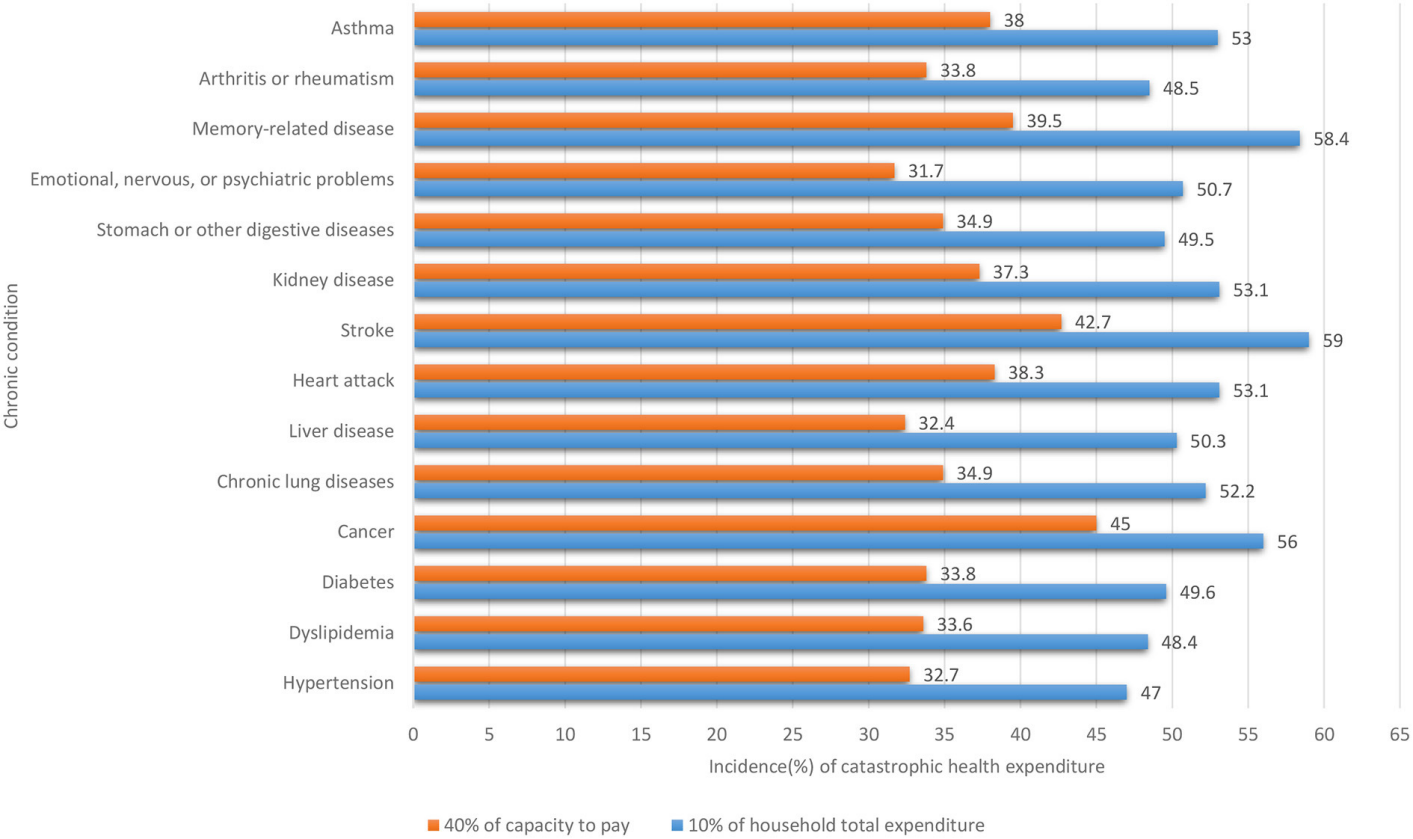
Incidence of catastrophic health expenditure by chronic conditions reported by respondents (*n* = 3511).

The incidence of CHE varied by the chronic conditions reported by the respondents. Regardless of the threshold adopted, the highest incidence of CHE was found in those who reported cancer, memory-related disorder, and stroke (Figure 1).

### Determinants of catastrophic health expenditure

Sex (p = 0.001), age (*p* < 0.001), marital status (*p* = 0.005), education (*p* < 0.001), number of chronic conditions (*p* < 0.001), outpatient visits over the past month (*p* < 0.001), inpatient admissions over the past year (*p* < 0.001), health insurance (*p* = 0.003), residency (*p* < 0.001), household size (*p* < 0.001), geographic location (*p* = 0.004), and socioeconomic status (*p* < 0.001) were all found to be associated with the incidence of CHE in the univariate statistical analyses (Table 3).

**Table 3:** Incidence of catastrophic health expenditure (CHE) by sociodemographic characteristics (*n* = 3511).

**Characteristics**	**With CHE**	**Without CHE**	**χ^2^**	**P**
	***n*** **(%)**	***n*** **(%)**		
Sex			10.688	0.001
Male	632 (33.9)	1230 (66.1)		
Female	475 (28.8)	1174 (71.2)		
Age (Years)			28.923	<0.001
60–69	574 (28.0)	1477 (72.0)		
70–79	401 (36.9)	687 (63.1)		
≥80	132 (35.5)	240 (64.5)		
Marital status			7.765	0.005
Married	864 (32.8)	1771 (67.2)		
Else	243 (27.7)	633 (72.3)		
Education			20.668	<0.001
Illiterate	295 (26.6)	702 (70.4)		
Primary school	551 (34.7)	1039 (65.3)		
Secondary school	255 (29.2)	617 (70.8)		
University	6 (11.5)	46 (88.5)		
Number of chronic conditions			94.421	<0.001
2	292 (23.6)	944 (76.4)		
3	266 (29.5)	637 (70.5)		
4	216 (35.4)	395 (64.6)		
≥5	333 (43.8)	428 (56.2)		
Hospital admissions over the past year			295.160	<0.001
Yes	274 (69.4)	121 (30.6)		
No	833 (26.7)	2283 (73.3)		
Outpatient visits over the past month			179.901	<0.001
Yes	243 (60.9)	156 (39.1)		
No	864 (27.8)	2248 (72.2)		
Health insurance			18.281	0.003
Basic medical insurance for urban employees	125 (24.9)	377 (75.1)		
Basic medical insurance for urban and rural residents	154 (36.2)	271 (63.8)		
New rural cooperative medical scheme	732 (32.7)	1509 (67.3)		
Basic medical insurance for urban residents	44 (28.0)	113 (72.0)		
Others	21 (25.6)	61 (74.4)		
None	31 (29.8)	73 (70.2)		
Residency			13.826	<0.001
Rural	898 (33.1)	1814 (66.9)		
Urban	209 (26.2)	590 (73.8)		
Household size			59.454	<0.001
≤ 2	863 (35.6)	1563 (64.4)		
>2	244 (22.5)	841 (77.5)		
Geographic location			13.413	0.004
Eastern	274 (27.9)	709 (72.1)		
Central	324 (31.3)	710 (68.7)		
Western	405 (33.1)	818 (66.9)		
Northeastern	104 (38.4)	167 (61.6)		
Per capita non–health household expenditure			146.185	<0.001
Quartile 1 (Lowest)	391 (44.5)	488 (55.5)		
Quartile 2	312 (35.5)	567 (64.5)		
Quartile 3	237 (27.1)	639 (72.9)		
Quartile 4 (Highest)	167 (19.0)	710 (81.0)		

The multivariate logistic regression models demonstrated good discriminatory validity (AUC = 0.777, 95% CI: 0.761 to 0.793) and calibration ability (Hosmer-Lemeshow χ^2^ = 7.790, *p* = 0.454). The results showed that CHE incidence increased by the number of chronic conditions reported by the respondents (aOR = 1.293–1.855, *p* < 0.05) and decreased with increasing household economic status (aOR = 1.622–4.595 relative the highest quartile, *p* < 0.001). Hospital admissions over the past year (aOR = 6.707, 95% CI: 5.186 to 8.674) and outpatient visits over the past month (aOR = 4.891, 95% CI: 3.822 to 6.259) of the respondents were the strongest predictors of CHE incidence. The respondents who were male (aOR = 1.266, 95% CI: 1.054 to 1.521), married (OR = 1.502, 95% CI: 1.211 to 1.862), older than 70 years (aOR = 1.288–1.458 relative to 60–69 years, *p* < 0.05), completed primary (aOR = 1.328 relative to illiterate, 95% CI: 1.079 to 1.635) or secondary school education (aOR = 1.305 relative to illiterate, 95% CI: 1.002 to 1.701), lived in a small (≤2 members) household (aOR = 2.207, 95% CI: 1.825 to 2.669), and resided in the northeast region (aOR = 1.935 relative to eastern, 95% CI: 1.396 to 2.682) were more likely to incur CHE. Health insurance (*p* = 0.065) and urban-rural residency (*p* = 0.663) were not significant predictors of CHE incidence after controlling for other variables (Table 4).

**Table 4:** Predictors of incidence of catastrophic health expenditure: Results of logistic regression model (*n* = 3511).

					**95% Confidence interval**	
**Independent variable**	**P-value**	**B**	**SE**	**aOR**	**Lower**	**Upper**
**Sex (Ref. female)**						
Male	0.012	0.236	0.094	1.266	1.054	1.521
**Age (Ref. 60–69 years)**	0.005					
70–79	0.007	0.253	0.094	1.288	1.071	1.548
≥80	0.011	0.377	0.148	1.458	1.090	1.948
**Marital status (Ref. else)**						
Married	<0.001	0.407	0.110	1.502	1.211	1.862
**Education (Ref. illiterate)**	0.003					
Primary school	0.008	0.284	0.106	1.328	1.079	1.635
Secondary school	0.049	0.266	0.135	1.305	1.002	1.701
University	0.050	−0.996	0.507	0.369	0.137	0.998
**Number of chronic diseases (Ref. 2)**	<0.001					
3	0.019	0.257	0.110	1.293	1.043	1.603
4	<0.001	0.491	0.121	1.634	1.288	2.073
≥5	<0.001	0.618	0.114	1.855	1.483	2.320
**Hospital admission over past year (Ref. No)**						
Yes	<0.001	1.903	0.131	6.707	5.186	8.674
**Outpatient visit over last month (Ref. No)**						
Yes	<0.001	1.587	0.126	4.891	3.822	6.259
**Health insurance (Ref. none)**	0.065					
Basic medical insurance for urban employees	0.538	−0.191	0.311	0.826	0.449	1.519
Basic medical insurance for urban and rural residents	0.664	0.116	0.267	1.123	0.665	1.896
Basic medical insurance for urban residents	0.698	−0.135	0.348	0.874	0.442	1.727
New rural cooperative medical scheme	0.315	−0.247	0.245	0.781	0.483	1.264
Others	0.162	−0.567	0.405	0.567	0.256	1.256
**Residency (Ref. urban)**						
Rural	0.663	0.080	0.184	1.084	0.755	1.555
**Household size (Ref**. **>2)**						
≤ 2	<0.001	0.792	0.097	2.207	1.825	2.669
**Geographic location (Ref. eastern)**	0.001					
Central	0.551	0.067	0.113	1.070	0.857	1.335
Western	0.074	0.195	0.109	1.215	0.982	1.503
Northeastern	<0.001	0.660	0.167	1.935	1.396	2.682
**Per capita non–health household expenditure (Ref. quartile 4)**	<0.001					
Quartile 1	<0.001	1.525	0.137	4.595	3.513	6.009
Quartile 2	<0.001	1.113	0.135	3.043	2.335	3.965
Quartile 3	<0.001	0.484	0.132	1.622	1.251	2.102

## Discussion

Multimorbidity is a significant risk of CHE. In this study, we found that 31.5% (≥40% OOP in CTP) to 45.6% (≥10% OOP in THE) households of old people (≥60 years) living with multimorbidity incurred CHE. This is lower than the CHE incidence (56.6%) revealed in another national representative study for households with diabetic patients with multimorbidity (23), even when our study participants were restricted to those with diabetes. The different results are a reflection of varied thresholds adopted: a more relaxed threshold (≥20% OOP in CTP) was adopted in the above-cited study. Nevertheless, it is clear that the households of old people with multimorbidity have a much higher incidence of CHE compared with the national average of 8.7% using the threshold of ≥40% OOP in CTP (39). We also found that household CHE incidence increases with the number of reported chronic conditions: in the households with a respondent reporting five or more chronic conditions, the odds of household CHE almost doubled that of those reporting only two chronic conditions. Empirical studies show that multimorbidity can lead to higher OOP health spending in both LMICs and high-income countries (12, 18, 40, 41).

Household incidence of CHE varies by the type of chronic conditions. We found that cancer, stroke, and memory-related diseases are the top three chronic conditions resulting in CHE: cancer ranked in top one using the threshold of ≥40% OOP in CTP, while stroke ranked in top one using the threshold of ≥10% OOP in THE. These results are consistent with the findings of other studies. Cancer is one of the most economically burdensome diseases, perhaps due to repeated outpatient visits and hospitalizations and rare and expensive drug treatments (42). Similarly, the need of continuous rehabilitation care for stroke patients also imposes a heavy financial burden on households (43). In many countries with aged populations, memory-related disease conditions such as dementia have resulted in rapid growth of health and care burdens (44, 45).

Socioeconomic and regional disparities in CHE incidence persist in China, according to the findings of this study and others. We found that the households with higher household economic status have significantly lower odds of incurring CHE. This is a common phenomenon, not just for the households with old people living with multimorbidity (13, 23, 35, 46, 47). Income inequality may exacerbate catastrophic health spending in the households with low income (39, 46). China has prioritized central financial subsidies to the least developed western region in tackling socioeconomic disparity concerns (48). However, the developing regions receiving less central subsidies (such as the northeast region) may be exposed to a higher risk of CHE as is indicated by the findings of this study. Interestingly, the central and western regions are not significant in the regression results. There are regional differences in the allocation of health resources in China, which may lead to lower access to quality health services in the central and western regions (49). Low access to health services can lead to unmet health service needs of patients, thus not generating higher OOP health expenditures (50, 51). As our results show, health-seeking behavior is the most significant determinant of catastrophic health expenditure.

It is interesting to note that the urban-rural disparity in CHE incidence is insignificant after controlling for other variables in our study. Rural households usually have lower income, enjoy lower welfare entitlements, and have lower access to quality health services than their urban counterparts (52, 53). This may lead to under use of healthcare services by rural residents (54). Indeed, our study participants were predominantly from rural households and their hospitalization rate is relatively low compared with the national average (55). Under use of healthcare services can lead to underestimation of CHE.

Health insurance is often considered as an important means of protecting individuals and families from CHE (56). However, it can be a double-edged sword if the funding pool is low, which can stimulate healthcare use without necessarily providing an adequate shield for CHE (13, 23). Some previous studies have found varied roles of different health insurance schemes in protecting households from CHE (47). However, we did not find any significant effects of the different insurance programs in their role of protecting households from CHE in comparison with those without any insurance. The new rural cooperative medical scheme has long been criticized for its limited role, if any, in alleviating household poverty induced by medical expenses (57). Although the integration of health insurance for urban and rural residents may help reduce the urban-rural disparity (53, 58), the overall level of insurance entitlements remains to be low in comparison with the basic medical insurance for urban employees (26).

We also found that male respondents and those who were married, older, completed primary or secondary school education, and lived in a small (≤2 members) household were more likely to incur CHE. These factors are usually labeled as predisposing factors for healthcare services use (13, 59). For example, education can improve health literacy and encourage use of healthcare services (60). A larger household size is usually associated with higher awareness of care needs and higher capacity to pay for medical services (39, 47).

China faces a great challenge in preventing CHE as its population is continuing to grow older while household size is shrinking (4, 39). The health system reform should pay increasing attention to care for the old people living with multimorbidity. The current level of protection offered by the existing health insurance programs is inadequate, despite a high coverage (13, 26). Healthcare services should strengthen primary care, which has a function of preventing avoidable hospital care (61). It is important a adopt a patient-centered approach in primary care, in particular for those living with multimorbidity as care for one condition may influence the others. Meanwhile, socioeconomic disparities need to be addressed.

## Limitations

Our study has some limitations. Firstly, data were collected through self-reporting, which may lead to underestimation of the prevalence of multimorbidity. The study participants may have some undiagnosed conditions, in particular in those living with low socioeconomic status. Secondly, the study did not assess severity of the chronic conditions due to unavailability of data. For example, sometimes a serious illness may cost more than two or more mild illnesses. Thirdly, the expenditure data are subject to recall bias. Fourthly, the study used a cross-sectional design. No causal relationships should be assumed.

## Conclusions

Multimorbidity is a significant risk of CHE for households of old people in China. CHE incidence increases with the number of reported chronic conditions. Socioeconomic and regional disparities in CHE incidence persist. The health insurance programs have not played a role in preventing CHE in older people with multimorbidity.

## Data availability statement

Publicly available datasets were analyzed in this study. This data can be found here: http://charls.pku.edu.cn/.

## Ethics statement

The studies involving human participants were reviewed and approved by the Biomedical Ethics Review Committee of Peking University (IRB0000105IRB00001052–11015). The patients/participants provided their written informed consent to participate in this study.

## Author contributions

Providing guidance for the overall content: WH, HY, and CL. Study design: WH, HL, EC, and WZ. Data analysis: HL, EC, and WZ. Writing and revising: WH and HL. Providing constructive suggestions for revisions: HY, CL, JL, EC, WZ, BL, JX, LZ, and WG. Embellishment: CL. Funding acquisition: WH and HY. All authors read and approved the final manuscript.

## Funding

Support for this study was provided by National Natural Science Foundation of China (72274045, 71974048) and China Medical Board (CMB-19-308).

## Conflict of interest

The authors declare that the research was conducted in the absence of any commercial or financial relationships that could be construed as a potential conflict of interest.

## Publisher's note

All claims expressed in this article are solely those of the authors and do not necessarily represent those of their affiliated organizations, or those of the publisher, the editors and the reviewers. Any product that may be evaluated in this article, or claim that may be made by its manufacturer, is not guaranteed or endorsed by the publisher.
